# Pilot study on the use of basophil activation tests and skin tests for the prevention of allergic transfusion reactions

**DOI:** 10.3389/falgy.2023.1328227

**Published:** 2024-01-08

**Authors:** Philippe Akiki, Laurence Dedeken, Alina Ferster, Virginie Doyen, Gwendy Dupire, Carole Nagant, Julie Smet, Nathalie Ghorra, Isabelle Ruth, Maïlis Lauwers, Valery Daubie, Francis Corazza, Hanane El Kenz

**Affiliations:** ^1^ Blood Bank Department, Brugmann University Hospital, Université Libre de Bruxelles, Brussels, Belgium; ^2^ Department of Pediatric Hematology Oncology, Hôpital Universitaire des Enfants Reine Fabiola, Université Libre de Bruxelles, Brussels, Belgium; ^3^ Department of Immuno-Allergology, Brugmann University Hospital, Université Libre de Bruxelles, Brussels, Belgium; ^4^ Laboratory of Immunology, Brugmann University Hospital, Université Libre de Bruxelles, Brussels, Belgium

**Keywords:** basophil activation test, skin tests, allergic transfusion reactions, transfusion medicine, sickle-cell disease, exchange transfusion

## Abstract

**Background and objectives:**

Management of severe allergic transfusion reactions (ATR) is challenging. In this study, we investigate the usefulness of skin tests and basophil activation tests (BAT) in chronically transfused patients for the prevention of future ATR.

**Materials and methods:**

BAT and skin tests were carried with the supernatant of red blood cell (RBC) units for a sickle-cell disease patient under chronic exchange transfusion who has presented a severe ATR, in order to prevent potential future ATR. If the results for both BAT and skin tests were negative, the RBC units could be transfused to the patient. If either one of the results was positive, the tested RBC unit was discarded for the patient.

**Results:**

192 RBC units were tested with both tests. The level of results concordance between the two tests was 95%. Out of the 169 negative units with both tests, 118 units were transfused to the patient for which he presented no ATR.

**Conclusion:**

In our study, combining both BAT and skin tests was associated with a good negative predictive value since we were able to safely transfuse our patient. Further studies are still necessary to confirm this result but this pilot study indicates that skin tests and BAT might help prevent ATR. When BAT is not available, skin tests may also be useful in preventing ATR.

## 1 Introduction

Management of severe allergic transfusion reactions (ATR) is challenging for clinicians and blood banks (1). ATR are common and most of them are usually mild presenting as urticarial lesions, pruritus and rashes but they can also present as localized angio-edema and, less frequently, as severe anaphylaxis (2–5).

Mast cells and/or basophils mediate these reactions. They are part of type I hypersensitivity reactions but in most cases the trigger and the exact pathophysiology of ATR remain uncertain (5, 6). Two pathways can intervene in ATR:

- IgE-dependent pathway: Allergens candidates among blood components may include plasma proteins (IgA, haptoglobin, C3, C4,…) in patients deficient in those proteins or chemical products such as methylene blue. There are no reliable estimates regarding the incidence of plasma proteins induced ATR, and chemical products ATR since such cases are rarely identified. Some reports have suggested that food allergens in blood components could play a role in ATR, but clear evidence has yet to be established (4, 6–9). These triggers may induce IgE bridging on the Fc*ε*RI and activate degranulation of mastocytes and/or basophils.
- IgE-independent pathway: Bioactive substances called biological response modifiers (BRMs) such as activated complement components, cytokines and chemokines accumulate in blood components during storage and are thought to directly activate mast cells/basophils via specific receptors. The exact mechanisms remain unsettled but it is possible that these BRMs induce anaphylactic reactions (2, 5, 8, 9).

Laboratory testing can help establish the causative relationship between the reaction and the transfusion.

Plasma protein levels and plasma protein antibodies against IgA and haptoglobin should be investigated (9).

Histamine is the primary mediator of anaphylaxis: its increase reflects the activation of mast cells during immediate hypersensitivity reactions (IgE and non-IgE mediated). However, plasma histamine can be difficult to measure (approximate half-life: 20 min) (10). Tryptase is the most abundant secretory granule-derived serine protease contained in mast cells (9). Its increase during mast cell activation is consistent with systemic anaphylaxis and other immediate hypersensitivity reactions. Although not as common, tryptase levels can also be elevated in non-IgE immediate hypersensitivity reactions. In routine practice, serum tryptase levels are measured at *T* < 4 h and *T* ≥ 24 h after transfusion in patients with suspected allergic transfusion reactions. After anaphylaxis onset, tryptase serum levels peak after approximately 15 min–120 min then decline with a half-life of approximately 2 h (3, 8–10).

However, tryptase has a couple of shortcomings: although its plasma half-life is longer than histamine, it is still short and pre-analytical conditions are sometimes difficult to respect. Moreover, a normal tryptase level does not rule out an IgE-mediated anaphylactic event. If the tryptase level is elevated during the reaction, a follow-up level should be obtained at a later time point to rule out an underlying mast cell disorder (3, 9, 10).

The Basophil activation test (BAT) was developed for understanding and managing allergic diseases but its day-to-day application in transfusion is still limited. Its sensitivity and specificity in blood transfusion remain to be determined (8). This test uses flow cytometry to assess basophil activation in sensitized patients via the upregulation of cell degranulation and the subsequent expression of activation markers (CD63 or CD203c) on the membrane of basophils when an allergen is incubated with whole blood. It can help determine the culprit allergen. Moreover, it could be used in patients with a known history of ATR to prevent future reactions.

Besides laboratory testing, an allergy investigation should be conducted. Skin tests are routinely used to test allergens or drugs. However, they are rarely used with blood components to prevent potential future ATR.

To our knowledge, currently there is no gold standard for proactively evaluating the potential for allergic transfusion reactions.

In this study, we carried out basophil activation testing and skin testing with the supernatant of red blood cell (RBC) units in order to prevent potential future ATR with the tested units. This study aims to investigate the contribution of skin tests and BAT in transfusion medicine and particularly in patients with continued chronic transfusion needs. Given that BAT is not always available, we also tried to establish if skin tests could be used as an alternative. This will allow us to establish a diagnostic protocol for the prevention of ATR in patients with a history of severe ATR.

## 2 Materials and methods

### 2.1 Recruitment of patients

The applicable patient population for an algorithmic evaluation of blood products similar to the one proposed in our protocol includes patients with history of at least one prior anaphylactic allergic transfusion reaction with transfusion needs.

Participation was proposed to all patients with transfusion needs that previously presented a severe ATR to any blood component at our hospitals (Brugmann University Hospital and Hôpital Universitaire des Enfants Reine Fabiola).

For this pilot study (from August 2020 to July 2022), only one patient met our criteria and was recruited. More patients are being included for an extensive study.

The patient is a 25 years old man with sickle-cell anaemia under regular chronic exchange transfusion since the age of 12 for frequent vaso-occlusive crises and acute chest syndromes despite good adherence to hydroxyurea. He experienced anaphylactic shock during an exchange transfusion of RBC units with severe hypotension, bradycardia, pruritus, urticaria and nausea/vomiting. Tryptase measures at *T* = 1 h (5.53 µg/L) and *T* = 24 h (1.58 µg/L) clearly favoured an immediate hypersensitivity reaction. Prior to this episode, the patient had never presented an ATR with any blood component. Extensive history-taking and multiple tests were done by allergists. Additional in-vitro (multiplex specific IgE tests) and in-vivo (skin tests) tests showed sensitization to grass and profilin. IgA and haptoglobin levels were within normal range.

### 2.2 Protocol

RBC units were tested with BAT and skin tests over a period of 2 years according to the patient's needs for transfusions or exchange transfusions.

Both tests were carried out with the supernatant of RBC units on the patient before transfusion, to prevent potential future ATR with the tested units.

For the BAT, supernatants of ABO, RH, Kell compatible RBC units were incubated as the potential allergen with the basophils of the patient. Allergists also used those supernatants for skin tests.

When the results for both tests were negative, the RBC units could be transfused to the patient. If either one of the results (BAT or skin tests) was positive, the tested RBC unit was excluded and not transfused.

The institutional ethics committee of Brugmann University Hospital and Hôpital Universitaire des Enfants Reine Fabiola approved this study (B0772023000001).

### 2.3 Peripheral whole blood

Blood samples were collected on anticoagulated (EDTA) peripheral whole blood and used for the BAT as a source of basophils.

### 2.4 Red blood cell units

The supernatant of compatible RBC units were obtained by centrifugation (5 min at 1,900 g) of units tubing. All the manipulations were carried out in a sterile manner.

### 2.5 Basophil activation test

BÜHLMANN Flow CAST KIT (BÜHLMANN Laboratories AG, Switzerland) was used to perform the BAT. For each RBC unit, 50 µl of the supernatant were mixed with 100 µl of Stimulation Buffer and 50 µl of the patient's whole blood. Then 20 µl of Staining Reagent were added. The tubes were gently mixed and incubated for 15 min at 37°C. Simultaneously, the same supernatant was also tested with the whole blood of a healthy control. For each BAT a negative control (containing 50 µl of Stimulation Buffer instead of the supernatant) and two positive controls (respectively containing 50 µl of anti-FcɛRI Ab and 50 µl of fMLP instead of the supernatant) were also prepared.

At the end of the first incubation, 2 ml of pre-warmed (20°C) Lysing Reagent were then added to each tube and mixed gently. This mix was incubated for 5–10 min at 20°C then centrifuged for 5 min at 500 g. The supernatant was eliminated and the cell pellet resuspended in phosphate-buffered saline. To ensure a constant time from blood collection to the BAT, the acquisition on the flow cytometer was performed within 4 h of blood collection for all samples.

**Figure 1:**
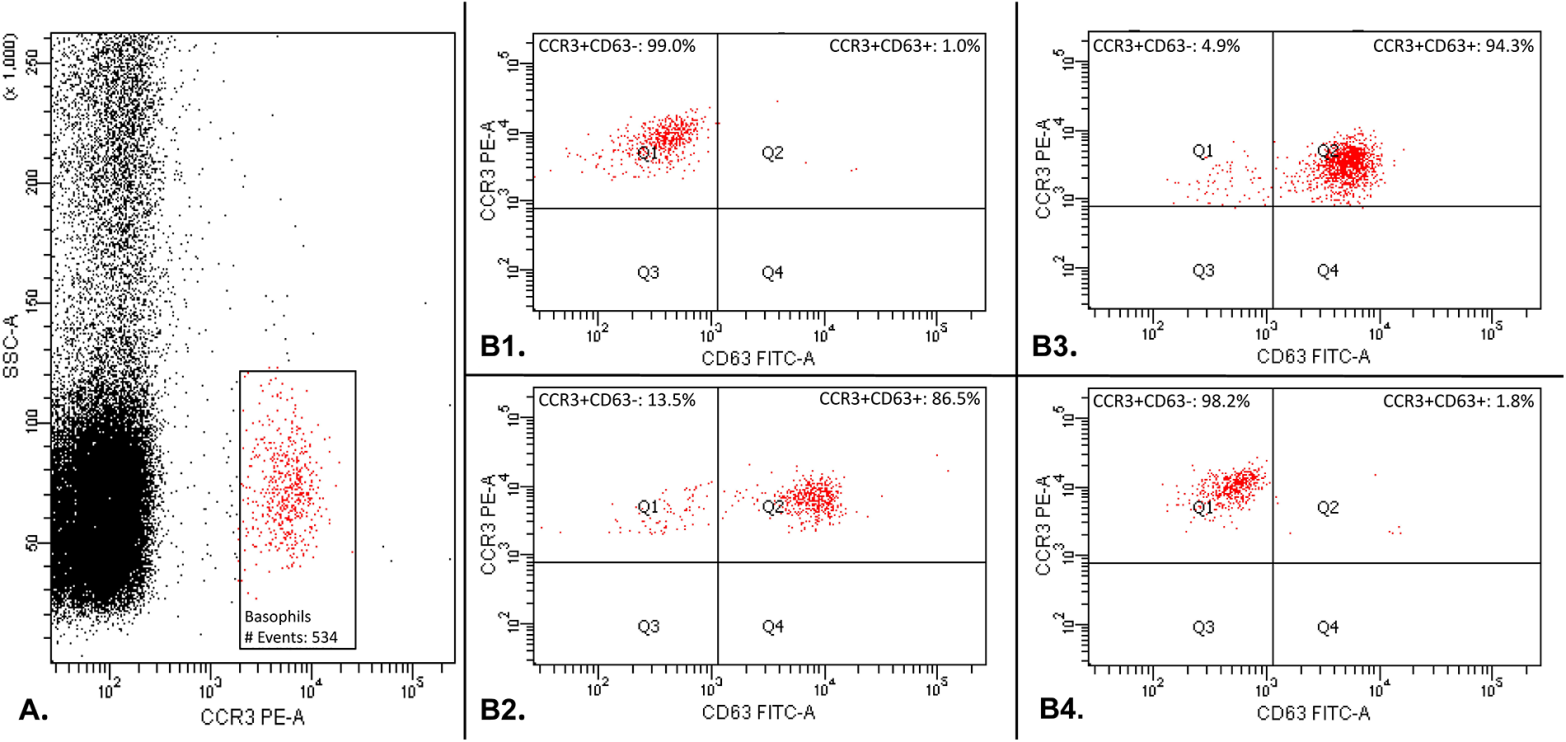
Basophil activation test gating: basophil population was gated by selecting cells that are CCR3_pos_ with a low side scatter (SSC-A_low_) (part A.). Secondly, CD63 was used as a basophil activation marker (Part B.). The percentage of CD63 positive cells was compared to the total amount of basophilic cells gated during the first step. Part B1 shows the result of the basophil activation test with a negative control in which the patient's basophils do not express the activation marker CD63. Part B2 shows the result of the basophil activation test with a positive control in which the basophils express the activation marker CD63. Part B3 shows an example of one RBC unit tested positive with the basophil activation test: the patient's basophils express the activation marker CD63. Part B4 shows an example of one RBC unit tested negative with the basophil activation test: the patient's basophil do not express the activation marker CD63.

Flow cytometric acquisition was performed on FACSCanto II (BD Biosciences, Erembodegem, Belgium). Acquisition was considered acceptable when a minimum of 500 basophils were collected. Gating is represented in Figure 1. The number of CD63 positive cells was expressed as a percentage of the total amount of basophils. A stimulation index similar to the one recommended by Hoffmann et al. was used as cut-off (11). The test was considered positive for a RBC unit when the patient's percentage of CD63 positive basophil exceeded 3 times the baseline percentage of CD63 postive basophil in the negative control.

### 2.6 Skin tests

We carried out percutaneous testing [skin prick-test (SPT)] followed by intradermal test (IDT). Depending on the number of tested units in the set, skin tests were done on either the forearm or the back of the patient according to the standard recommendations. For SPT, we introduced the skin device at a 90° angle into the upper layer of the skin, through a drop of the supernatant of a RBC unit. Histamine (5.43 mmol/L) was used as a positive control and physiological serum as a negative control. Results were read after 15 min and the test was considered positive if the patient had a wheal ≥3 mm with a localized erythema (12). For IDT, 0.02 ml of diluted supernatant (0.1 ml of allergen in 0.9 ml of physiological serum) was injected into the dermis. Results were read after 20 min and the test was considered positive if the patient presented a wheal ≥3 mm with an erythema than the one caused by the injection (12).

### 2.7 Statistical analysis

BAT data was tested for normalised distribution using D’Agostino-Pearson. As distribution was not parametric, data was analysed using the Mann–Whitney *U*-test. Statistical significance was defined as *p* < 0.05. BAT mean values, standard error, 95% confidence interval, median values, 25% and 75% percentile were also given as descriptive statistics. Statistical analysis were performed using GraphPad Prism version 8.0.1 for Windows (GraphPad Software, San Diego, California, USA).

## 3 Results

192 RBC units were tested in the adequate conditions with BAT, SPT and IDT. All SPT results were negative. BAT and IDT results are summarized in Table 1. The level of concordance between BAT and IDT was high (95%). The discrepancy between both tests only occurred 9 times for the 192 units tested.

**Table 1:** Contingency table with BAT and IDT results for the tested RBC units.

		BAT		Total IDT
		Positive	Negative	
IDT	Positive	14	8	22
	Negative	1	169	170
Total BAT		15	177	192

Using the Mann–Whitney *U*-test, BAT median value in the positive group was significantly higher than the BAT median value in the negative group (*p* < 0.0001). Descriptive statistics are summarized in Table 2.

**Table 2:** Descriptive statistics for BAT results.

	RBC units with negative BAT	RBC units with positive BAT
Number	177	15
Mean (% basophils CD63+) [95% Confidence Interval]	1.55 (1.30–1.80)	44.83 (25.14–64.52)
Standard error	0.13	9.18
Median (% basophils CD63+)	1.10	38.80
25%–75% Percentile	0.70–1.60	13.10–90.00

Over a two-year period, the patient underwent 26 transfusions and/or exchange transfusions for which 119 RBC units were used according to the protocol. All the procedures went well without clinical reaction except once: due to a miscommunication between clinicians, one positive unit in IDT and negative in BAT was transfused although it should have been excluded according to protocol. The patient rapidly reacted to this unit (after 110 ml of transfused RBC) despite the premedication (5 mg of oral Levocetirizine and 250 mg of Hydrocortisone IV). He presented localized pruritus followed by generalized pruritus. The transfusion was immediately stopped and the patient received an additional 5 mg of oral Levocetirizine. No further signs or symptoms were observed. Tryptase levels at *T* = 1 h (4.81 µg/L) and *T* = 24 h (1.58 µg/L) were in favour of an ATR.

## 4 Discussion

Multiple hemovigilance reports indicate that ATR is one of the most reported incident related to transfusion. According to the French national hemovigilance report in 2021, the incidence of ATR was 17.9/100,000 transfused blood components or 10/10,000 patients transfused. Severe ATR are less frequent (1/100,000 transfused blood component) than mild reactions (4). However, they can present a heavy burden on blood banks and their management can be challenging.

Previous studies set forth the utility of BAT as a functional test in transfusion medicine by using healthy individuals and patients with a history of ATR (Table 3) (2, 5, 13–20). It was used to assess the causal relationship between transfusion and ATR and to examine the mechanisms of the reaction (2, 5, 13, 21). However, in this study, we tried to use the BAT in combination with skin tests to prevent potential future ATR.

**Table 3:** Review of studies using BAT in transfusion medecine.

Article	BAT		Results	Deduced BAT possible utility
	Source of basophils	Tested allergen		
Matsuyama et al. (6)	5 HV	9 SN of PC associated w/ATR in other patients	3/9 BAT(+) w/basophils of ≥1 HV	- Ability of SN fr. ATR cases to activate basophils of HV → NCM BAT - Assess the potential risk of the involved BP to cause ATR in other patients
		12 SN of PC not associated w/ATR in other patients	0/12 BAT(+) w/basophils of HV	
Matsuyama et al. (13)	5 HV	37 SN of PC associated w/ATR in other patients	3/37 BAT(+) w/basophils of 1 HV	- Ability of SN fr. ATR cases to activate basophils of HV → NCM BAT - Assess the potential risk of the involved BP to cause ATR in other patients
			3/3 BAT inhibited by Dasa.	- Assess ATR pathway: IgE-dependent vs. IgE-independent
			BAT in favor of fish allergens in one donor	- Assess the suspected Ag.
Nubret et al. (14)	Patient 1	FFP-MB fr. same donor & MB	BAT(+) w/FFP-MB & BAT(−) w/MB	- Ability of SN to activate the patient’s basophil → CM BAT - Assess the suspected Ag.
	Patient 2	MB	BAT(+)	
Dewachter et al. (15)	Patient	MB	BAT(+)	- Ability of SN to activate the patient’s basophil → CM BAT - Assess the suspected Ag.
Iwamoto et al. (16)	Patient^a^	Haptoglobin	BAT(+)	- Ability of Haptoglobin to activate the patient’s basophil - Assess the suspected Ag. - Assess ATR pathway: IgE-dependent vs. IgE-independent
Okamura et al. (2)	Patient 1	1 SN of PC associated w/patient 1 ATR	1/1 BAT(+) w/o Dasa. & (−) w/Dasa.	- Ability of SN to activate the patient’s basophil → CM BAT - Assess the potential risk of ATR in future transfusions - Assess ATR pathway: IgE-dependent vs. IgE-independent
		3 SN of PC associated w/ATR in other patients	0/3 BAT(+)	
	Patient 2^b^	1 SN of PC associated w/patient 2 ATR	1/1 BAT(+) w/o Dasa. & (−) w/Dasa.	
		12 SN of PC associated w/ATR in other patients	8/12 BAT(+)	
	2 HV	2 SN of PC associated w/patient 1 & 2 ATR	0/2 BAT(+) w/basophils of HV	
Yasui et al. (17)	2 Patients^b^	SN of the corresponding PC for each patient	2/2 BAT(+) w/o Dasa. & (−) w/Dasa.	- Ability of SN to activate the patient’s basophil → CM BAT - Assess ATR pathway: IgE-dependent vs. IgE-independent
	2 HV	2 SN of PC associated w/patient 1 & 2 ATR	0/2 BAT(+) w/basophils of HV	
	Quasi-basophils^c^ with: IgE fr. 2 patients or IgE fr. HV	SN of the corresponding PC for each patient	2/2 pi-BAT(+) w/IgE fr. 2 patients sera 0/2 pi-BAT(+) w/IgE fr. HV sera	- Ability of SN to activate quasi-basophils with IgE fr. patients - pi-BAT: assess ATR in patients undergoing myelo-suppression
Yasui et al. (18)	Quasi-basophils^c^ with : IgE fr. 9 non-ATR patients or IgE fr. 22 patients^d^	SN of the corresponding BP for each patient	0/9 pi-BAT(+) in non-ATR & 3/12 pi-BAT(+) in mild ATR & 10/10 pi-BAT (+) in Mod. to Sv. ATR - Mod. to Sv. ATR vs. non-ATR: Cut-off point: 7.9% & Sensitivity: 0.9 & specificity: 1.0	- Ability of SN to activate quasi-basophils with IgE fr. patients - pi-BAT: assess ATR in patients undergoing myelo-suppression
Yasui et al. (19)	5 HV	28 SN of PC associated w/ATR 15 FFP associated w/ATR	BAT(+) w/1 FFP when incubated w/4 HV w/o Dasa. & (−) w/Dasa. → Identified IgG anti-IgE in this FFP, which was characterized using BAT and pi-BAT w/16 additional HV.	- Assess ATR pathway: IgE-dependent vs. IgE-independent - Assess the suspected Ag. - Assess the potential risk of the involved BP to cause ATR in other patients - pi-BAT may help assess the suspected Ag
Usami et al. (20)	27 patients w/ATR/FNHTR	41 BP associated w/ATR 5 BP associated w/FNHTR 37 BP not associated w/ATR/FNHTR	Median BAT in ATR BP: 22.1%; in FNHTR BP: 27.8%; in non-ATR/FNHTR BP: 8.5%. BAT values comparable regardless of ATR severity & BAT(−) w/Dasa.	- Ability of SN to activate the patient’s basophil → CM BAT - Assess ATR pathway: IgE-dependent vs. IgE-independent - Assess the potential risk of ATR - Assess the causal relationship between BP and ATR/FNHTR
	27 patients w/ATR/FNHTR 19 patients w/o ATR/FNHTR	34 BP associated w/ATR 37 BP not associated w/ATR/FNHTR	BAT values in ATR/FNHTR patients >BAT values in non ATR/FNHTR patients even with BP not associated w/ATR/FNHTR	
	9 HV	1 BP associated w/ATR 1 BP associated w/FNHTR 1 BP not associated w/ATR/FNHTR	No differences in BAT results between BP	

In this table, “patient” was used for patients with a History of ATR unless otherwise specified.

ATR, allergic transfusion reaction; Ag., antigen; BAT, basophil activation test; BP, blood-products; CM, cross-matched; Dasa: dasatinib; FFP, fresh-frozen-plasma; FNHTR, febrile-non-hemolytic-transfusion-reaction; From, fr.; HV, healthy volunteer; IDT, intradermal test; MB, methylene-blue; Mod., moderate; NCM, non-cross-matched; PC, platelet concentrate; pi-BAT, passive immune basophil activation test; Sv., severe; SN, supernatants; w/, with; w/o, without.

^a^ Anhaptoglobinemia with IgG anti-haptoglobin (+) and IgE anti-haptoglobin not detectable.

^b^ Patient 2 of Okamura et al. (2016) and patient 1 of Yasui et al. (2017) are the same case.

^c^ The term Quasi-basophils was used here to refer to basophils stripped fr. their own surface IgE.

^d^ 12 mild and 10 moderate to severe ATR.

Out of the 169 units negative with both tests, 118 units were safely transfused to the patient and 51 units were unneeded. The patient did not present any reaction to those transfusions, which suggest a high negative predictive value when combining both tests. This is an encouraging result but the performances (sensitivity, specificity, positive predictive values…) of the tests need to be assessed in future studies with a methodology similar to Yasui et al. (18). It would consist of testing with BAT and skin tests residuals from blood products associated with ATR and residuals from blood products not associated with ATR.

Furthermore, in transfusion medicine, skin tests are not sufficiently studied and hence rarely used. Therefore, we also aimed to investigate the utility of skin tests as a possible alternative to BAT in transfusion medicine, by analysing concordance between those tests, since BAT is not always available.

The level of results concordance between the BAT and IDT in this study was high (95%). However, when analysing the discordant results, we noticed that 8/22 units were positive in IDT and negative in BAT, and only 1/15 unit was positive in BAT but negative in IDT. This is possibly related to the difference of sensitivity and specificity of each test. Nevertheless, since positive units (with BAT and/or IDT) were not transfused considering the risk, the sensitivity and specificity of both tests could not be determined. However, a new ATR occurring after the accidental transfusion of a RBC unit positive for IDT and negative for BAT suggests that IDT might have a higher sensitivity than BAT since IDT was positive and BAT was negative for this RBC unit. Further studies with both BAT and IDT are needed to establish sensitivity and specificity for each test.

If those results are confirmed in further studies, an algorithm for patients with transfusion needs and a history of severe ATR could be established: we would evaluate multiple units with both tests and only transfuse the units with negative results for both tests in order to minimize the risk of ATR. When BAT is unavailable skin tests might be tested alone to improve the management of severe ATR.

Washing of blood components can also be useful to reduce ATR (22, 23). However, this procedure comes at a cost of product damage, higher risk of infections and a reduced shelf life. BAT and skin tests are then to be considered as another protection layer.

This might be useful especially since systematic reviews and meta-analysis studies suggest that premedication remains not evidence based (24–26).

This study has several limitations. Since positive units were not used as a challenge test because of the clinical risk for the patient, we could not establish sensitivity and specificity of both tests.

Secondly, we did not find a consensus in the literature on cut-off values for BAT in transfusion medicine (27). This should be addressed in future studies.

Moreover, this study included only one patients. More patients need to be included to confirm these results. However, it is important to note that for this single patient, 192 RBC units were tested adequately and 118 transfused safely.

Finally, as it is often the case in ATR, we were unable to identify a common allergen among the positive units.

In summary, this pilot study using BAT and skin tests illustrates their application in transfusion medicine, particularly their possible utility in preventing ATR. Further prospective studies are necessary to confirm these results and establish a diagnostic protocol for the prevention of ATR in patients with a history of severe ATR.

## Data Availability

The data is not publicly available due to ethical restrictions. Requests to access the datasets should be directed to philippe.akiki@lhub-ulb.be.
